# Skin gluten-related disorders: new and old cutaneous manifestations to be considered

**DOI:** 10.3389/fmed.2023.1155288

**Published:** 2023-05-17

**Authors:** Alice Verdelli, Alberto Corrà, Elena Biancamaria Mariotti, Cristina Aimo, Lavinia Quintarelli, Valentina Ruffo di Calabria, Marta Elettra Donati, Veronica Bonciolini, Emiliano Antiga, Marzia Caproni

**Affiliations:** ^1^Department of Health Sciences, Rare Dermatological Diseases Unit, Azienda USL Toscana Centro, European Reference Network-Skin Member, Florence, Italy,; ^2^Section of Dermatology, Department of Health Sciences, University of Florence, Florence, Italy; ^3^Dermatology Unit, San Jacopo Hospital, Pistoia, Italy

**Keywords:** gluten-related disorders, skin extra-intestinal manifestations, coeliac disease, dermatitis herpetiformis, wheat allergy, non-celiac gluten sensitivity

## Abstract

The term gluten-related disorders (GRD) refer to a spectrum of different clinical manifestations triggered by the ingestion of gluten in genetically susceptible individuals, including coeliac disease (CD), wheat allergy and non-celiac gluten sensitivity (NCGS). GRD are characterized by a large variety of clinical presentations with both intestinal and extra-intestinal manifestations. The latter may affect almost every organ of the body, including the skin. Besides the well-known association between CD and dermatitis herpetiformis, considered as the cutaneous specific manifestation of CD, many other muco-cutaneous disorders have been associated to GRD. In this review, we analyzed the main features of dermatological diseases with a proven association with GRD and those that improve after a gluten-free diet, focusing on the newly described cutaneous manifestations associated with NCGS. Our main hypothesis is that a “cutaneous-gluten sensitivity,” as specific cutaneous manifestation of NCGS, may exist and could represent a diagnostic marker of NCGS.

## Introduction

The term gluten-related disorders (GRD) refer to a spectrum of diverse clinical manifestations triggered by the ingestion of gluten in predisposed patients (1). GRD include: (1) autoimmune reactions, e.g., coeliac disease (CD), dermatitis herpetiformis (DH) and gluten ataxia; (2) IgE-mediated allergy, e.g., wheat allergy (WA) and 3) immune-mediated non-coeliac gluten sensitivity (NCGS) (2).

GRD are characterized by both intestinal and extra-intestinal manifestations, including neurological, cutaneous, reproductive, and musculoskeletal manifestations (3). Although the extra-intestinal manifestations of GDR are ascertained, their prevalence remains poorly established (4).

The evidence of correlation between skin and bowel disorders in both children and adults is supported by a large amount of literature in this regard. Several muco-cutaneous diseases related to gluten intake other than DH are increasingly reported (5–8). Despite improvements in understanding the pathogenic aspects of GRD, the mechanisms underlying the onset of dermatological diseases still remain unclear (9). The most probable hypotheses rely on the loss of immunotolerance in genetically predisposed individuals as well as on increased bowel permeability which would enable the release of gluten related peptides leading to autoimmune response, vascular alterations and subsequent vitamin and aminoacidic malabsorption (8–9). In this review, the main aspects of dermatological disorders associated with GRD and the response to a gluten-free diet (GFD) are described. Particularly, we focused on the newly described cutaneous manifestations associated with NCGS.

## Coeliac disease

CD is a chronic autoimmune systemic disease triggered by gluten intake, which affects genetically predisposed individuals of both sexes of any age. The overall prevalence of CD is about 0.5–1% (10). The pathophysiological aspects of CD involve genetic, environmental and immunological factors. Indeed, in genetically predisposed patients, the gluten or gluten-related proteins intake can induce immunological responses with bowel inflammation and tissue damage. Specific T cell populations, pro-inflammatory cytokines, autoantibodies against an intestinal enzyme, and impaired T regulatory cells are involved in the intestinal damage (11). Moreover, alteration of the gut microbiome (“dysbiosis”) may play a relevant role into CD pathogenesis, driving the gluten mediated immune response. However, it is difficult to establish whether the CD-associated dysbiosis is a consequence, a simple concomitant association or a concurring causative factor (12–16).

The diagnosis of CD relies on a combination of genetic, clinical features, serology and histology.

Human leukocyte antigen (HLA) DQ2 (>90%) or HLA DQ8 (5–10%) is present in almost all patient with CD (7). The HLA risk alleles is an essential factor for the development of CD, with an high negative predictive value (17).

The assessment of circulating IgA anti-tissue transglutaminase 2 (TG-2) antibodies is the most sensitive serological test for CD, although deamidated gliadin peptide IgG antibodies might be useful in seronegative patients with innate IgA deficiency (16, 17–20). Endoscopic biopsy provides relevant information demonstrating characteristic histologic features ranging from lymphocytic infiltration, crypt hyperplasia to various degrees of villous atrophy (18, 19). While most of the guidelines available allow the diagnosis of CD without biopsy in children under strict conditions (19), endoscopy with duodenal biopsies is still mandatory to achieve a final diagnosis in adults (21). In children with anti-TG-2 positivity lower than 10 times the upper limit, at least 4 biopsies from the distal duodenum and at least 1 from the bulb should however be taken (19). Recently, Roca et al. (22) suggested the detection intestinal anti-TG2 deposits as a complementary or supporting tool for CD diagnosis in pediatric population with symptoms suggestive of CD and low-grade histological picture.

CD is characterized by a wide spectrum of symptoms. Intestinal manifestations include diarrhea, abdominal distention, constipation, and malabsorption (23). Extra-intestinal symptoms, which can present as first sign of CD, include weight loss, weakness, anemia, reduced bone mineral density, recurrent aphthous stomatitis (RAS), hypertransaminasemia, musculoskeletal pain, spontaneous abortions, epilepsy, peripheral neuropathy and dermatological lesions, reflecting the systemic nature of the disease (24, 25).

### CD and skin diseases

CD has been associated with several diseases (26–28) including skin disorders, in both adults and children (25, 29–32).

The relationship between CD and skin disorders is intriguing and has been confirmed in a recent paper by Lebwohl et al. (33). They investigated 43,000 celiac patients and 198,000 controls and found that patients with CD are at increased risk of multiple common skin disorders compared to the general population. However, the reasons of these associations are not clear yet and should be investigated in further studies, but probably include shared genetic risk factors and biological mediators related to CD itself. To explore a common background between skin manifestations and CD, many genetic studies have been conducted on patients with psoriasis (34, 35). For example, a recent meta-analysis reported the overlap of 10 psoriasis susceptibility loci with those of CD, including a single nucleotide polymorphism (rs6822844) which is strongly associated with CD and both psoriasis and psoriatic arthritis (36). Moreover, it is of note that the two conditions share immunogenic mechanisms, since both psoriasis and CD are T-cell mediated diseases (37). Although CD is considered a Th2- mediated disease, immunologic studies showed an important role of Th1, Th17, and Tγδ cells in both CD and psoriasis. In consideration of the altered intestinal barrier in CD, it may be assumed that an increased permeability to immunogenic triggers may lead to a higher prevalence of immune-mediated disorders.

Recently, Humbert et al. (38) proposed a classification of CD associated skin diseases by dividing them in four categories: autoimmune, allergic, psoriasis and miscellaneous. In addition, sporadic associations with other skin diseases were subsequently reported (9).

DH is currently considered the specific cutaneous manifestation of CD (39). However, many other dermatoses have been reported in coeliac patients (7, 8, 23) including psoriasis, atopic dermatitis (AD), urticaria, RAS, rosacea and vitiligo (40).

According to a recent meta-analysis, psoriatic patients appear to have a three-fold increased risk of CD, with an odds ratio (OR) of 3.09 (95% confidence interval [CI]: 1.92–4.97) (41). Moreover, a meta-analysis by Bhatia et al. (37) demonstrated a higher rate of anti- gliadin IgA antibodies positivity in patients with psoriasis compared to the controls (OR: 2.36, 95%CI: 1.15–4.83) with a potential role of GFD in controlling psoriasis on those patients. Other studies suggested a correlation between levels of CD antibodies and psoriasis or psoriatic arthritis severity (42, 43).

Studies on AD showed a higher prevalence and incidence of autoimmune conditions, including CD, both in adults and children (44–47). Risk of new-onset autoimmune disease was doubled especially in people with severe AD compared to controls (44).

In a recent study by Shalom et al. (48) on 116.816 patients (of whom 45.157 adults), AD was.

associated with a significantly higher prevalence of CD (OR: 1.609, 95%CI: 1.42–1.82, *p* < 0.001). The frequency of AD was significantly higher in a pediatric celiac population compared to ulcerative colitis or Crohn’s disease (49). Moreover, Ress et al. (50) found a four-fold greater risk of developing CD in children with AD. Finally, in a case–control study (51) on 4.114 adult patients, AD frequency was found to be three-fold higher in CD patients and two-fold in their relatives than in their spouses.

Autoimmune diseases, including CD, have been linked also to chronic urticaria (CU) (52). Kolkhir et al. (53) observed a strong link between chronic idiopathic urticaria and various autoimmune diseases, including CD, with a reduction of flares after a GFD. Case reports have described patients with CD who develop CU following gluten ingestion (54–56).

In a meta-analysis by Nieri et al. (57) authors demonstrated higher incidence of RAS in CD patients (OR: 3.79, 95%CI: 2.67–5.3). Yılmaz et al. (58) found a significant higher prevalence of RAS compared to the control group. This association has mostly been studied in pediatric populations. It is not known whether RAS lesions are directly influenced by a gluten sensitivity disorder, or if they are related to low levels of serum iron, folic acid, and vitamin B12 or trace element deficiencies due to malabsorption in patients with untreated CD. Local and systemic conditions, immunological and microbial factors as well as oral dysbiosis may also have a pathogenic role (59).

An association between CD and rosacea has also been demonstrated (60). One study showed a higher risk of CD in females with rosacea (61). In a nationwide cohort study, the prevalence of CD was higher among patients with rosacea when compared to controls (62).

The association between CD and vitiligo is still debated (63). Some authors (64–66) found a higher incidence of vitiligo in CD patients, while Volta et al. did not find any correlation between these two immune diseases (67). Other hypothesized associations between skin disease and CD (7, 8, 23) include lupus erythematosus (68), dermatomyositis (69), Behçet disease (70), pemphigus (71, 72), linear IgA bullous dermatosis (73), and lichen (74, 75). Rarely, prurigo nodularis (76), cutaneous vasculitis (7, 21), necrolytic migratory erythema (77), erythema nodosum (78), pityriasis lichenoides (79), porphyria (80), cutaneous amyloidosis (81), pityriasis rubra pilaris (82), erythroderma (83), partial lipodystrophy (84),generalized acquired cutis laxa (85), ichthyosis (86), atypical mole syndrome and congenital giant nevus (87) have also been described.

Most of these associations may be considered coincidental, such as the association between CD and alopecia areata (AA), with prevalence in CD patients similar to the one of the general population (88). AA sometimes improves or disappears with a GFD, but the efficacy of the diet could be explained by a non-specific regularization of immune response (8).

Secondary intestinal malabsorption can also cause muco-cutaneous manifestations due to nutrients deficiency. Zinc deficiency in CD patients has been associated to crusty-erythematous-squamous dermatitis localized in to periorificial regions, genitals and folds. Moreover, diffuse alopecia, stomatitis, balanitis, vulvitis, and proctitis were also found in these patients (8).

Iron deficiency could be associated with atrophy, xerosis, itching, hair loss, atrophic glossitis, angular stomatitis, and koilonychias while pytiriasis rubra pilaris-like lesions were found in vitamin A deficiency. Lower vitamin B12 and folic acid serum levels have been associated to angular stomatitis, glossitis, oral ulcers, and hyperpigmentation (8). Finally, in the literature, we found a case of pellagra associated with CD (89).

Concerning oral involvement, CD patients could be affected by both dental and oral mucosa anomalies including enamel defects, RAS, delayed tooth eruption, multiple caries, angular cheilitis, atrophic glossitis, dry mouth and burning tongue (90). In a recent meta-analysis (91), patients with CD showed a significantly higher prevalence of enamel defects compared to healthy people. Zoumpoulakis et al. (92) observed a higher prevalence of enamel defects and aphthous-like ulcers in CD patients. Cruz et al. (93) found a significant association between CD and enamel defects and dry mouth in a pediatric population, but this association was not confirmed for RAS. Recently, Lambertet al (94) reported an association between perineal basal cell carcinoma in patients with long-standing untreated CD, suggesting that CD may predispose patients to develop skin malignancies in this region, possibly due to chronic exposure of perineal skin to inflammatory molecules from the gastrointestinal tract. More data are needed to support this association, however inspection of all mucous membranes, including the anal mucosa, is important when CD is suspected.

There are no specific guidelines for the diagnosis and treatment of skin diseases associated with CD, excluding DH (95). The suspicion of a possible association is mainly based on a careful history and on the persistence or worsening of the skin lesions despite the standard of care treatment and requires specific dermatologic capability. Histologic, immunopathologic, serologic examinations as well as other investigations (for example patch test) could help making a correct diagnosis. GFD is mandatory in all the patients and could be helpful in solving skin lesions in association with current treatment guidelines.

## Dermatitis herpetiformis: typical and atypical manifestations

DH, specific skin manifestation of CD, affects approximately the 13% of patients with CD, while the highest prevalence of DH has been reported in Finland with 75 estimated cases per 100.000 people (96, 97). However, DH incidence seems in reduction, differently from the four-fold increase detected in the incidence of CD (98). DH is typical of adulthood; however DH can be also diagnosed in childhood and in adolescence (99–101). While a female prevalence is reported in CD, previous studies on DH showed a male to female ratio ranging from 1.1 to 1.9 (102). Epidermal transglutaminase (TG-3), rather than TG-2, is the main autoantigen in DH and it may explain why skin manifestations appear in a proportion of patients having gluten sensitive disease (103, 104). Our group of work found a higher rate of positive antibody testing in females, confirming similar results of previous studies, while serologic testing was found to be less sensitive for male patients with CD (102).

The pathogenic mechanism underlying DH is multifactorial, involving genetic, environmental, and immunologic factors (105). In susceptible individuals with hidden CD with a TG-2, and possibly also a TG-3 autoantibody response, the development of skin lesions may result from immune complex deposition in the wall of dermal vessel, by complexes of high avidity anti-TG-3 IgA antibodies together with the TG-3 enzyme (104, 106–108).

Polymorphous presentation with characteristic distribution of skin lesions is the clinical hallmark of DH (109). Primary DH lesions consist of grouped erythematous papules and/or urticarial plaques surmounted by vesicles or blisters (110), with variable severity of the rash. In our experience, blisters were detectable with a higher frequency among patients aged more than 65 while elderly patients are more prone to severe itching and therefore to a higher frequency of scratching lesions compared to pediatric patients. These results could partially be explained by the higher degree of dryness of elderly skin (102). Due to the intense itch and scratching, rupture of the blisters occurs and often only erosions, crusts are present (111). Chronic pruritus and excoriations may lead to lichenification while a transient post-inflammatory hyperpigmentation may occur after resolution.

Typically, the lesions develop symmetrically on the extensor surfaces of the elbows (90%), knees (30%), shoulders, middle line of the back, buttocks, and sacral area. Occasionally other sites are involved, such as the scalp, face, upper back and neck (5, 110).

The diagnosis is based on typical clinical picture and immunopathological findings (95, 112, 113). According to the European Guidelines, direct immunofluorescence (DIF) performed on a sample of perilesional skin is the gold standard for the diagnosis (95). The pathognomonic finding is represented by granular IgA deposit with an arrangement which may assume different patterns: granular deposits at the tips of dermal papillae, granular deposits along the dermal-epidermal junction (DEJ),or a combination of both configurations (95). In up to 50% of Japanese patients, a fibrillar deposition of IgA has been described (114).

Histopathological analysis of lesional skin biopsy is not mandatory for diagnosis, as the findings are not invariably specific for DH (95). The most typical histopathological features consist of subepidermal vesicles and blisters with neutrophilic infiltration at the papillary tips. Eosinophils can be found within the inflammatory infiltrate in some cases. Small bowel mucosal biopsies are not mandatory for DH diagnosis either: even though the severity of the mucosal damage varies between patients with DH, it seems not to have any effects on the long-term prognosis (111).

While no circulating autoantibodies specific for cutaneous basement membrane components or to other adhesion structures are detected, DH patients present gluten-induced IgA autoantibodies targeting TG-2 and TG-3 (108, 115). Only IgA-class TG-2 antibody-based serological immunoassays can be helpful, such as indirect immunofluorescence (IIF) microscopy and enzyme-linked immunosorbent assay (ELISA) (115). TG2 positivity alone is not sufficient for the diagnosis of DH as false positivity may occur, even if it has a high positive predictive value. Conversely, TG2 negativity does not exclude the possibility of DH (95).

Apart from the classic findings in DH, several atypical cases were reported in literature (110, 116). An uncommon skin manifestation is represented by asymptomatic palmo-plantar purpuric lesions, alone or in association with the characteristic findings, describe in children more often than in adults (117–120).

Naylor et al. (121) published a DH patient presented with diffuse petechial rush and microscopic changes consistent with both DH and vasculitis. Kern et al. (122) described a case of DH presenting as pseudovasculitis with diffuse petechial rush and an ulcer on forearm, and histopathology and DIF were both compatible with DH, with no signs of vasculitis. Finally, atypical clinical presentation of DH includes palmoplantar keratosis (123), wheals of chronic urticaria (124) and lesions mimicking prurigo pigmentosa (125).

In all these studies the immunopathological findings were supportive, resulting in inclusion of such skin lesions in the spectrum of DH. However, a granular IgA deposit along the DEJ was found also in healthy skin of patients with CD (126, 127) as well as in the perilesional skin of CD patients with other skin diseases different from DH (128), suggesting the role of such deposits not only as marker of DH, but, more generally, of CD. In this view, all the previous defined atypical DH may be considered part of the skin spectrum associated to CD, more than a real DH.

Thus, DH diagnosis should be conceived as the result of an overall assessment, including clinical manifestations, compatible histological findings and granular IgA immunopathological detection.

DH lesions usually resolve with GFD. Pasternack et al. (129) showed that more than one-third of patients with DH on GFD showed prolonged symptoms, lasting up to 2 years after being diagnosed, and 14% after long- term dietary treatment. More severe rash at diagnosis was associated with the persistence of skin symptoms, and those having skin symptoms despite long-term GFD had a shorter duration and more relapses on their diet. Thus, careful monitoring of these patients is essential and in case of persistence of symptoms, a systemic therapy with dapsone may be associated. Signs and symptoms of DH usually resolve within 3–4 days of starting dapsone. The starting dose can be either low or high depending on the severity of the skin. Other symptomatic therapy options, such as sulfasalazine, potent topical corticosteroids and antihistamines, are significantly less efficient and may be considered only if dapsone is contraindicated, not tolerated or the patient does not give consent to its use (95).

## Wheat allergy

WA is defined as an adverse IgE mediated immunologic reaction to wheat. IgE autoantibodies recognize specific epitopes of allergens, triggering an inflammation cascade (11).

Depending on the way the subject is exposed to allergens and to the underlying immunologic mechanisms, WA may manifest as food allergy affecting the skin, gastrointestinal tract, or respiratory tract; food-dependent, exercise-induced anaphylaxis (FDEIA); occupational asthma (so- called baker’s asthma) and rhinitis; or contact urticarial (130).

WA prevalence, both in children and adults is approximately 1% (0.4–4%), depending on the age of the patients and the regions taken under consideration. When considering the patients with food allergies, WA is diagnosed in 11–20% of children and in 25% of adults (131, 132). Children have a higher.

prevalence of WA compared to adults, especially if wheat is introduced in the diet for the first time after 6 months of age. The increased prevalence in children compared to adults can be explained by the fact that most of the patients outgrow their allergy by the age of 16 years (132).

WA may manifest with a variety of symptoms including urticarial/angioedema, asthma, allergic rhinitis, abdominal pain, vomiting, acute exacerbation of AD and wheat-dependent exercise-induced anaphylaxis (WDEIA). All these symptoms may start within 2 h after the first exposure to wheat (133) .

The majority of children allergic to wheat appear to suffer from moderate-to-severe AD and wheat ingestion may elicit typical IgE-mediated reactions (e.g., urticarial/angioedema, bronchial obstruction, nausea and vomiting, abdominal pain, systemic anaphylaxis (134)) while in adults, an allergic reaction to ingested wheat is infrequent. The most common variant is the WDEIA, where symptoms result from the combination of causative food intake and physical exercise (as well as non-steroidal anti-inflammatory drugs or alcohol). Xu et al. (135) analyzed 193 patients with a diagnosis of wheat-induced anaphylaxis (WIA), 104 of whom presented WDEIA and 177 recurrent urticaria, including 12 wheat- induced urticaria. Their results indicated that 6.8% of patients with recurrent urticaria were allergic to wheat, and they had a potential risk of WIA/WDEIA. In this population gastrointestinal symptoms could be mild and difficult to recognize, being mainly represented by diarrhea and bloating (134). Moreover, the adult-onset of WA seems to be a transient form of food allergy with a very favorable prognosis. In a cohort of 13 adults patients affected by WA prospectively followed for a definite period of time, WA resolved in 90% of cases after a mean period of 4.2 years (136).

In a recent study by Faina et al. (137) on 176 WA patients, cutaneous manifestations were found in 69.3% of cases. Among them, urticarial/angioedema was the most common one (42%) followed by eczematous dermatitis (41%) including AD, chronic hand eczema and generalized eczematous dermatitis. Rarely, generalized itching (9%) and psoriasiform lesions (8%) were also described. Moreover, WA patients sensitized to grasses reported a worsening of cutaneous manifestations after grain ingestion and during the pollen season, with a prevalence in aero-exposed body areas.

In a study on 179 adolescent and adult patients, Čelakovská et al. (138) observed a correlation linking WA and exacerbation of AD in 4.5% of adult patients.

In WA several proteins can act as allergens and the WHO/IUIS Allergen Nomenclature Database (139) describes 21 different wheat allergens in the latest version. Some of them seem mainly associated with respiratory symptoms such as the alpha-amylase/trypsin inhibitor proteins, while non-specific lipid transfer protein (nsLTP) and gliadins seems to be associated to food allergy. WDEIA seems to be correlated to omega-5 gliadin protein while contact urticaria with high molecular weight (HMW) glutenins. However, clinical manifestations induced by different proteins may overlap (134).

Tammaro et al. (140) found eczematous lesions on the face, neck, and arms of 14 celiac patients, after application of gluten-containing hygiene products, or after contact with foods containing wheat and durum wheat. Five of them resulted positive to patch-by-patch with wheat in correspondence of their sites of application. Authors hypothesized that a subgroup of celiac patients could develop, after repeated contact with gluten, a real contact allergy or a hypersensitivity reaction to this allergen or to other wheat derivates but their study was conducted on a small sample.

In conclusion, the diagnostic process for WA requires the evaluation of the clinical history, a complete physical examination and further analysis in selected cases. It should be considered in individuals with anaphylaxis or skin manifestations that manifest within minutes to hours after food intake, especially in children, or in the case of second episode after specific food ingestion (141, 142). First-line investigations include the measurement of specific wheat extract IgE, wheat allergens IgE and skin prick test (SPT). The detection of specific IgE without a clear history of symptoms occurred after wheat exposure should not be considered diagnostic as many people tolerating wheat exposure can be sensitized to wheat, especially grass pollen sensitive individuals (134). Indeed, patients with grass pollen sensitivity often produce IgE specific for cereal derived allergens, due to common epitopes in wheat flour and grass pollen which can induce the production of cross-reacting IgE (143).

In the case of ingestion-induced wheat allergy in paediatric and adult patients, the clinical history and the results of the aforementioned tests can be completed with oral food challenge, for which double-blind placebo- controlled challenge remains the gold standard (144). The diagnosis of WDEIA should include patient’s history and examinations (allergy skin tests, wheat-specific IgE basophil activation test, histamine release test and/or exercise challenge test) (145).

Primary treatment and management of WA is avoidance of both food and inhaled wheat allergens. In cases of anaphylactic reaction, the administration of epinephrine is the lifesaving treatment. All patients should go to the emergency room for further evaluation and antihistamines, glucocorticoids, and beta-agonist are additional secondary treatment options. Additional treatments include immunotherapy such as oral immunotherapy (OIT), sublingual immunotherapy (SLIT), and epicutaneous immunotherapy (EPIT). OIT and SLIT both utilize the principle of gradually increasing the amount of food ingested leading to progressive desensitization and thus hopefully helping to avoid experiencing an allergic reaction. EPIT utilizes a skin patch to deliver an allergen to the patient. Those with WDEIA require epinephrine and can prevent symptoms by avoiding exercising within four to six hours after wheat ingestion, avoid exercising alone or in hot weather/pollen season, and carrying emergency medication (146, 147).

## Non-coeliac gluten sensitivity

The NCGS is characterized by a cohort of symptoms related to gluten-containing food ingestion which improve following GFD in subjects not affected by CD or WA (148). Although the first cases of NCGS were reported in 1980 (149), this entity has been recently rediscovered by Sapone et al. (2) who focused on clinical and pathophysiological features of NCGS. Since then, the number of publications on NCGS has grown exponentially, in parallel with the number of patients treated with GFD for the large spectrum of symptoms (146–154). Despite its inclusion as a separate condition in the spectrum of GRD, NCGS shows some features resembling CD, i.e., immunological involvement and response to GFD, and some others typical of irritable bowel syndrome (IBS) (148).

Differently from CD, HLA-DQ2, or HLA-DQ8 characterize the genotype of only 50% of NCGS patients. Autoimmunity and IgE-mediated allergy seems not to be pathogenetic mechanisms implicated in this condition, while a possible role of the innate immune system has been proposed (12, 155, 156). However, the intestinal burden is variable, ranging from mild symptoms (157–160) to a more severe involvement with low QoL, as shown by Tovoli et al. (161) Of the 41 patients analyzed, Authors found higher intestinal (65.9%) and extraintestinal manifestations (72.7%) in NCGS patients in comparison with CD and patients still complain symptoms, even if significantly attenuated by the GFD, even years after the diagnosis.

An impaired intestinal epithelial barrier has also been demonstrated *in vivo* and *ex vivo* studies (162).

In addition, intestinal dysbiosis may contribute to the pathogenesis of NCGS enhancing epithelial barrier dysfunction and associated inflammatory response to gluten, similarly to what has been shown in CD and other inflammatory bowel diseases (163, 164). It is unclear whether gliadin is the real responsible of the disease, since other wheat component including amylase-trypsin inhibitors (ATIs), fermentable oligo-di-monosaccharides and polyols have been proposed as triggers (165). For this reason, some authors suggested to replace the term “NCGS” with “non-celiac wheat sensitivity,” highlighting that, apart from gluten, other potentially bioactive component of wheat and related cereals are also excluded in a GFD (166).

The prevalence of NCGS was not clearly defined yet. According to self-reported data, it ranges between 0.5 and 13% in the general population, with a higher prevalence in women, teenagers, and patients in their 3rd to 4th decade of life (167, 168). However, the lack of diagnostic biomarkers makes difficult to obtain such an estimation. Differently from CD and WA, NCGS still remains a diagnosis of exclusion (169), since no clear serologic or histopathologic criteria are currently available. The final diagnosis relies both on negative criteria (exclusion of CD and WA) and positive criteria (modifications of symptoms after gluten withdrawal and rechallenge in blind).

Recently, Kirmizi et al. (170) analyzed duodenal mucosae of 44 NCGS patients, reporting preserved villous architecture, normal or mildly increased intraepithelial lymphocytes (IELs) with clusters, with normal count of eosinophils and mast cells. They proposed the irregular distribution of IELs with clusters in the villous epithelium as an histological finding supporting the diagnosis of NCGS, but larger studies are needed to confirm their results. Up to date, the Salerno Experts’ Criteria (157) are the only accessible recommendations for diagnosis of NCGS. Autoantibodies such as anti-TG2 and anti-endomysial (EMA) IgA are usually negative in NCGS. Anti-gliadin IgG are detected in up to 50% of the patients, while anti-gliadin IgA rarely occur (7%) (134, 148).

The non-specific clinical presentation of NCGS includes gastrointestinal symptoms, such as abdominal pain, bloating or altered bowel habits, which may variably be associated with further systemic manifestations such as fatigue, headache, joint pain, mood disorders and skin lesions. The onset usually occurs after few hours or days after gluten consumption and symptoms disappear following gluten withdrawal (154, 155, 169).

To date few studies on the cutaneous manifestations associated with NCGS are available in literature. Carroccio et al. (171) found that one third of 276 NCGS patients with irritable bowelsyndrome-like symptoms were also affected by AD, while a subsequent study of the same group (172) showed an AD prevalence of 42% among the 60 NCGS patients examined, which also showed nickel allergy with contact dermatitis in 10% of the cases. Interestingly, patients with NCGS and nickel allergy had higher frequency of cutaneous manifestations after wheat ingestion than NCGS patients without nickel allergy (100 vs. 7%). Indeed, erythema following wheat ingestion appeared in all NCGS patients with nickel allergy, whereas diffuse itching and urticaria were observed in 50 and 33% of them, respectively. In comparison, only 10% of NCGS patients without nickel allergy showed cutaneous manifestations.

Volta et al. (173) conducted a prospective study on 486 NCGS suspected patients, demonstrating that more than 20% of subjects had an allergy to inhalants (26% to mites), food, or metals. The authors reported “skin rash” and “dermatitis” in 29 and 18% of patients, respectively; however, they did not better define the morphology of skin lesions.

Recently, our group studied 17 subjects with NCGS and associated skin lesions (174). In our experience, patients with NCGS showed non-specific, often itchy, dermatoses which clinically resembled DH in some cases, while other patients showed eczematous or psoriasiform manifestations. GFD seemed to induce significant improvement or clearance of skin manifestations. Lesions were represented mainly by erythematous papules and vesicles, often excoriated and extremely itchy, as in DH or subacute eczema. Some patients presented erythematous and hyperkeratotic plaques with excoriations, resembling chronic psoriasis. Skin lesions were present for over 1 year in 59% of patients, whereas in other patients the disease was longer with a typical relapsing course. Interestingly, DIF of perilesional skin showed deposits of the C3 fraction along the DEJ with a granular or micro-granular pattern in 82% of the patients. IgA deposits were detected in a non-significant proportion of cases, without a specific distribution pattern, thus differentiating NCGS from DH. Gastrointestinal symptoms mainly resembled IBS with abdominal pain, bloating, flatulence, diarrhea or constipation, with improvement after GFD, as well as for skin lesions which cleared up within a month in most of cases.

At the time, we hypothesized a “cutaneous gluten sensitivity” (CGS) characterized by itching skin lesions, C3 microgranular/granular deposits at the DEJ and a prompt response to GFD. Recently, we analyzed the DIF pattern of a total of 45 NCGS patients, finding C3 microgranular/granular pattern associated to a superficial perivascular C3 and/or IgM deposits in 86.6% of them (175).

In 2017, Faina et al. (137) studied 163 patients with NCGS, of whom 65.6% presented skin lesions. In adults, the incidence was higher among females, while this correlation was inverse in children. All enrolled patients showed gastrointestinal symptoms, represented by abdominal pain and meteorism in 80% of cases and diarrhea, constipation and/or nausea in 20% of patients. Skin lesions were described as eczematous in 45% of patients, urticaria/angioedema-like in 36.4% and psoriasiform in 9% of cases. Atopic dermatitis, chronic hand eczema (CHE), AD localized to the hands, palmar psoriasiform-dermatitis and generalized eczematous dermatitis were the most common forms. All the patients complained of intense itch, which was the only symptom in about 10% of cases. Authors did not report histological and DIF examinations, however all the patients showed response to GFD.

In a Japanese study (176), authors found 20 patients with DH-like clinical features, showing granular deposition of C3 with no IgA, IgG or IgM deposits along the DEJ at DIF. Their findings were different from those found in any known autoimmune bullous diseases or other inflammatory skin diseases. Moreover, there were no gastrointestinal symptoms suggesting CD or other gastrointestinal disorders, thus no patients underwent endoscopic studies for either upper or lower intestinal tracts. Authors proposed the term “granular C3 dermatosis” as a possible new disease entity for this condition. According to our findings (175), we hypothesized that these manifestations could be part of NCGS more than a new entity. Particularly, 23.5% of our 45 NCGS patients presented with DH-like lesions without gastrointestinal symptoms but a microgranular/granular C3 deposit at DEJ was present in all of them (complete data not previously shown). Clinically, the patients showed itchy dermatoses, resistant to traditional local and systemic therapies, which promptly solved following GFD. In 18.9% of NCGS patients a history of AD was found, while 10.8% patients showed nickel allergy.

According to our results, the introduction of the concept of specific skin manifestations associated with NCGS with peculiar DIF pattern may be helpful for patients’ management, as for DH and CD.

The treatment of NCGS involves the dietary restriction of the suspected triggers of the disease, but there is controversial data about the effectiveness of different dietary interventions such as GFD and low-FODMAP diet. Nevertheless, current evidence supports the GFD still represents first-line therapy. However, a FODMAP restriction can decrease gastrointestinal symptoms in individuals. Further research is needed to confirm this finding (177, 178).

## Conclusion

Besides the invariable association between DH and CD, other skin diseases represent common extra-intestinal manifestation of GDR. Identifying these conditions could reveal helpful, especially when resistant to standard therapy, since they may improve under GFD with no adjunctive therapies. Therefore, our considerations highlight the need of close cooperation between gastroenterologists and dermatologists.

Concerning gluten sensitivity, the skin is frequently involved among extra-intestinal NCGS manifestations, since patients often show itchy dermatoses with eczema-like, DH-like or psoriasis- like appearance. A distinct feature is represented by the DIF with C3 deposits along the DEJ with microgranular/granular pattern.

Clinical and immunological studies on larger populations of patients with NCGS could lead to the definition of a specific skin pattern, which itself may be sufficient to make a diagnosis of bowel disease, as for CD and DH. In fact, following the preliminary data reported above, the definition of clinical and immunopathological characteristics of the NCGS-associated skin manifestations is essential to define a new clinical entity with specific features, the so called CGS. Moreover, it may be of interest the characterization of the metabolomic profile and microbiota of both skin and gut of patients with NCGS. Dysbiosis in NCGS causes gut inflammation, diarrhea, constipation, visceral hypersensitivity, abdominal pain and dysfunctional metabolic state. As for the microbiota and the gastrointestinal tract, there is a symbiotic relationship between microbial communities and skin, it is possible to hypothesize that skin microorganisms may also influence the course of disease associated with extraintestinal manifestations. Thus, another goal for the future may be to characterize the metabolomic profile and skin and intestinal microbiota in course of NCGS, in order to identify specific markers for the diagnosis of CGS and, therefore, of NCGS itself.

## Capsule summary

We describe the main dermatological features of gluten-related disorders, focusing on the newly described cutaneous manifestations of non-celiac gluten sensitivity. The introduction of the concept of specific skin manifestations related to non-celiac gluten sensitivity to may be helpful for the management of these patients.

## Author contributions

AV, MC, EA, and VB contributed to conception of the study. AV, EBM, and AC wrote the first draft of the manuscript. EBM, LQ, CA, VR, and ME wrote sections of the manuscript. All authors contributed to the article and approved the submitted version.

## Conflict of interest

The authors declare that the research was conducted in the absence of any commercial or financial relationships that could be construed as a potential conflict of interest.

## Publisher’s note

All claims expressed in this article are solely those of the authors and do not necessarily represent those of their affiliated organizations, or those of the publisher, the editors and the reviewers. Any product that may be evaluated in this article, or claim that may be made by its manufacturer, is not guaranteed or endorsed by the publisher.
